# Prevalence of overweight and obesity among Iranian population: a systematic review and meta-analysis

**DOI:** 10.1186/s41043-023-00419-w

**Published:** 2023-07-24

**Authors:** Behnaz Abiri, Amirhossein Ramezani Ahmadi, Shirin Amini, Mojtaba Akbari, Farhad Hosseinpanah, Seyed Ataollah Madinehzad, Mahdi Hejazi, Amirreza Pouladi Rishehri, Alvand Naserghandi, Majid Valizadeh

**Affiliations:** 1 Obesity Research Center, Research Institute for Endocrine Sciences, Shahid Beheshti University of Medical Sciences, Tehran, Iran; 2 Isfahan Endocrine and Metabolism Research Center, Isfahan University of Medical Sciences, Isfahan, Iran; 3 Department of Nutrition, Shoushtar Faculty of Medical Sciences, Shoushtar, Iran; 4 Department of Nutrition, School of Public Health, Iran University of Medical Sciences, Tehran, Iran; 5 Student Research Committee, Shahid Beheshti University of Medical Sciences, Tehran, Iran

**Keywords:** Obesity, Overweight, Prevalence, Body mass index, Iran

## Abstract

**Background:**

Obesity is a major risk factor for chronic diseases. Politicians and practitioners should be aware of the dramatic increase in obesity and its subsequent complications to prevent associated health risks. This systematic review aimed to provide better insight into the prevalence of overweight and obesity in the Iranian population.

**Method:**

An evaluation was conducted on all published observational studies from both national (SID, Irandoc, Iranmedex) and international (Web of Knowledge, PubMed, Scopus) sources, which reported the prevalence of overweight/obesity among normal population samples, between January 2012 and December 2021.

**Result:**

A total of 152 eligible studies were included in this meta-analysis. Of the 152 selected studies, 74 reported the prevalence of overweight/obesity in patients aged ≤ 18 years, and 61 studies in adults. In the rest of the articles (17 studies), the results were reported for a combination of these age groups. The prevalence of overweight and obesity in Iran was estimated at 20.1 (95% CI 17.92–22.30) and 13.44 (95% CI 11.76–15.22), respectively. This percentage (95% CI) was 11.71 (10.98–12.46) for overweight and 8.08 (7.02–9.22) for obesity in those aged ≤ 18 years, and 35.26 (32.61–37.99) for overweight and 21.38 (19.61–23.20) for obesity in those aged > 18 years. The overall prevalence of overweight and obesity in the entire population was 35.09% (95% CI 31.31–38.98).

**Conclusion:**

As obesity is on the rise in Iran, we should seek both weight loss strategies and ways to control comorbidities associated with high BMI.

## Introduction

Obesity, one of the most significant public health concerns in the world, is physiologically characterized by the abnormal or excessive accumulation of fat within adipose tissue, which may cause serious health issues [1]. Globally, body mass index (BMI) is the most commonly used practical indicator of overweight and obesity [1]. There is growing evidence that obesity and overweight are increasing worldwide [2], with substantial differences in prevalence levels and trends between countries. The problem of excessive weight not only affects adults, but has also become a concern among children, even in developing countries [2].

It has been suggested that the increasing prevalence of obesity can be attributed to lifestyle changes, particularly dietary habits, and inadequate physical activity in both rural and urban settings [1]. Some ethnic dimensions can also contribute to obesity, including genetics, eating patterns, and socioeconomic status [1, 3].

Several studies have shown that obesity increases the risk of chronic and life-threatening illnesses, including type 2 diabetes, cardiovascular disease, hypertension, hyperlipidemia, and sleep apnea, and reduces life expectancy by approximately 7 years [4, 5]. According to the 2015 global burden of disease estimates, increased weight was responsible for at least 4.0 million deaths (7.1% of all deaths) and 120 million disability-adjusted life years (4.9% of all disability-adjusted life years) [6]. Moreover, we will face a major health problem in the near future due to obesity-related comorbid disorders that will require massive funding. At the same time, we will have limited resources [7, 8]. Thus, prevention and control of this risk factor is a public health priority, especially in developing countries such as Iran. To implement programs for primordial and primary prevention of noncommunicable diseases, health policymakers at national and international levels require insights into the prevalence of overweight and obesity.

Our meta-analysis represents a novel approach to estimate the prevalence of overweight and obesity in Iran. Although previous meta-analyses have focused on specific age or sex groups, none have investigated the prevalence of obesity in the entire Iranian population. To address this gap, our study took advantage of all available data on the topic, examining age and geographical distribution and using the most recently published reports from 2012 to 2021, which ensures that the results are accurate and up to date. By providing a more comprehensive analysis of obesity in Iran, our study offers new insights into the prevalence and distribution of obesity in the country. These findings have important implications for public health policies and practices in Iran, particularly for developing effective prevention and management strategies for the general population.

## Methods

Throughout this systematic review and meta-analysis, we collected all relevant studies reporting the prevalence of obesity and overweight among the normal population across all regions of Iran. In the following sections, we discuss the strategy of this study in detail.

### Search strategy

**Fig. 1:**
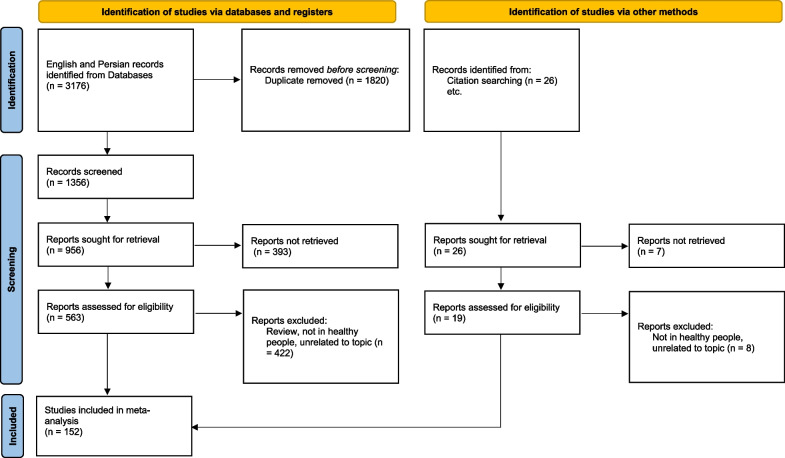
PRISMA flow diagram for selection process of the studies

A medical information specialist and review team collaboratively designed the electronic search strategies. We searched in English databases; Scopus, ISI web of Sciences, PubMed, and Google scholar and also in Persian databases; IranMedex, Scientific Information System (SID), and Irandoc collect all related studies, during the time period January 2012 through December 2021. The titles, keywords, and abstracts of all databases were evaluated. The medical subject headings (MESH) were: “overweight”, “obesity”, “Iran”, “body mass index”, and “prevalence” for searching in English databases, and for searching Persian databases, the equivalent Persian-language terms were used as well. In addition, a manual search was conducted to identify articles that were not found through an electronic search. Figure 1 illustrates the study selection process. The systematic review was performed according to the Preferred Reporting Items for Systematic Reviews and Meta-Analyses (PRISM) Statement [9]. Code of pre-registration of the systematic review and meta-analysis protocols in PROSPERO is 392744.

### Study selection

All related population-based studies, including national, provincial, and local surveys, were included, regardless of age or sex restrictions. We considered studies that (i) had an observational design, (ii) reported the prevalence of overweight and/or obesity, and (iii) used BMI as an indicator of overweight and obesity. In contrast, clinical trials, reviews, editorials, studies on nonhuman models, and those without access to the full text were excluded.

The extracted data of children and adults were recorded in two separate Excel sheets (Microsoft Office package 2010), including the name of the first author, year of publication, data collection, study design, study region, age of participants, number of sample sizes (total and sex), definition of obesity and overweight, and the reported prevalence of overweight/obesity.

### Data extraction and quality assessment

During the first and second steps, the titles and abstracts of the studies were assessed to determine whether they met the inclusion and exclusion criteria. Abstracts that were insufficient were then reviewed by reading their full texts. Since BMI as a conventional variable has been evaluated in many studies, we searched the full texts to determine the prevalence of obesity and overweight, even in cases where obesity or overweight was not the primary objective. Two researchers (BA and SM) independently performed all procedures. When there was no agreement, a consensus was reached through debate. Otherwise, a third expert reviewer (ARA) was asked to make a decision.

The Newcastle–Ottawa Scale (NOS) was used to assess the quality of observational studies [10]. The data extraction and quality assessments are presented in Table 1.

**Table 1:** General characteristics of retrieved studies (presented in chronological order, starting with the most recent)

First author (reference no.)	Year of publication/data collection	Study design	Province/city	Age (year)	Sample size (M/F)	Prevalence of overweight, obesity	Overweight/obesity definition	Study quality (NOS)
Amin [11]	2021/2016	Cross-sectional	All 31 provinces of Iran	> 18	28,321 (13,708/14,613)	37%, 23.2%	BMI 25.0–29.9/BMI > 30.0	5
Najafipour [12]	2021/phase 1:2009–2011/phase 2:2014–2018	Cross-sectional	Kerman/Kerman	15–80	Phase 1: 2820 (NM) Phase 2: 9997 (NM)	Phase 1: 33.5%, 15.4% Phase 2: 35.8%, 22.3%	BMI 25.0–29.9/BMI > 30.0	5
Sayadi [13]	2021/2019	Cross-sectional	Shiraz	40–70	7225 (3445/3780)	44.9%, 28.9%	BMI 25.0–29.9/BMI > 30.0	5
Mahdavi-Roshan [14]	2021/2014–2017	Cross-sectional	Guilan/Some’e Sara	35–70	10,520 (4887/5633)	39.9%, 32.7%	BMI 25.0–29.9/BMI > 30.0	5
Zokaei [15]	2021/NM	Cross-sectional	Alborz	≥ 30	2947 (707/2240)	51.7%, 35.4%	BMI 25.0–29.9/BMI > 30.0	5
Zoghi [16]	2021/2016–2018	Cross-sectional	Hormozgan	35–70	3917 (1691/2226)	78%, 46%	BMI 25.0–29.9/BMI > 30.0	4
Siervo [17]	2021/NM	Cross-sectional	Fars	35–70	4296 (1925/2371)	35.8%, 17.2%	BMI > 25/BMI > 30.0	5
Amininezhad [18]	2021/NM	Cross-sectional	Bushehr	68.94 ± 6.21	1342 (115/1227)	42.6%, 26.4%	BMI 25.0–29.9/BMI > 30.0	4
Azadi [19]	2021/NM	Cross-sectional	Southern Iran	40–75	874 (273/601)	46%, 25%	BMI 25.0–29.9/BMI > 30.0	4
Alami [20]	2021/2019	Cross-sectional	Khorasan razavi/Gonabad and Bajestan	15–64	1106 (553/553)	31.4%, 12.4%	BMI 25.0–29.9/BMI > 30.0	4
Mohammadi [21]	2021/2018–2019	Cross-sectional	Ardabil	9.61 ± 1.76	581 (286/295)	19.1%, 20.17%	BMI: 85th–95th, BMI > 95th	4
Hadianfard [22]	2021/2019	Cross-sectional	Yazd	2–16	510 (258/252)	Overweight and obesity: 40%	BMI ≥ 1SD	4
Nazari [23]	2021/NM	Cross-sectional	Lorestan	6–18	866 (419/447)	13%, 9.2%	BMI: 85th–95th, BMI > 95th	4
Taherifard [24]	2021/2018	Cross-sectional	Southern Iran	15–65	276 (114/162)	19%, 24%	BMI 25.0–29.9/BMI > 30.0	3
Jafari [25]	2021/2017	Cross-sectional	Yazd	≤ 1	602 (304/298)	7.3%, 3.5%	BMI: 85th–95th, BMI > 95th	4
Dastgheib [26]	2021/2016	Cross-sectional	Fars/Kharameh	40–70	8222 (3992/4226)	Overweight: 53.4%	BMI > 25.0	5
Entezarmahdi [27]	2021/2017	Cross-sectional	West Azerbaijan/NM	2–5	2432 (1228/1204)	Obesity: 1.4%	BMI > 2SD	4
Asoudeh [28]	2021/NM	Cross-sectional	Isfahan/Isfahan	36.8 ± 8.1	5219 (NM)	Obesity: 18.9%	BMI > 30.0	5
Najafi [29]	2020/2014–2017	Cross-sectional	14 Provinces in Iran	49.41 ± 9.18	129,257 (57,614/71,643)	40.76%, 30.43%	BMI 25.0–29.9/BMI > 30.0	5
Parvaresh [30]	2020/2014–2015	Cross-sectional	Kerman/Kerman	7–12	1017 (519/498)	13.7%, 14.3%	BMI: 85th–95th, BMI > 95th	5
Pourabdian [31]	2020/2013–2016	Cross-sectional	Isfahan	> 20	36,625 males	39.1%, 10.8%	BMI 25.0–29.9/BMI > 30.0	5
Djalalinia [32]	2020/2016	Cross-sectional	National	> 18	31,050 (NM)	59.3%, 22.7%	BMI > 25.0/BMI > 30.0	5
Taghdir [33]	2020/2004–2012	Cross-sectional	Fars/Shiraz	41.1 ± 10.6	11,850 females	41.4%, 24.1%	BMI 25.0–29.9/BMI > 30.0	5
Anjom‑Shoae [34]	2020/NM	Cross-sectional	Isfahan	18–55	8691 (3450/5241)	18.6%, NM	BMI > 25.0	5
Katibeh [35]	2020/2010–2011	Cross-sectional	Yazd	54.1 ± 10.0	2098 (1104/994)	41.8%, 26.7%	BMI 25.0–29.9/BMI > 30.0	5
Nabati [36]	2020/2017–2018	Cohort	South Caspian	> 18	1008 (606/402)	46.13%, 31.45%	BMI 25.0–29.9/BMI > 30.0	4
Shayan-Moghadam [37]	2020/NM	Cross-sectional	Isfahan	12–18	3207 females	11.3%, 10.2%	1SD < BMI < 2SD/BMI > 2SD	4
Abbaspoor [38]	2020/2018	Cross-sectional	Khuzestan/Ahvaz	30–65	600 females	36.5%, 36.5%	BMI 25.0–29.9/BMI > 30.0	4
Alamolhoda [39]	2020/2014	Cross-sectional	Fars/Shiraz	14–20	2538 (1286/1252)	10.2%, 5.1%	BMI: 85th–95th, BMI > 95th	4
Mozaffari‑Khosravi [40]	2020/2019	Cross-sectional	Yazd	12–16	569 (NM)	Overweight and obesity: 37.8%	BMI ≥ 1SD	4
Mardani [41]	2020/2017	Cross-sectional	Lorestan/Khorramabad	15–17	832 females	16.3%, 5.8%	BMI: 85th–95th, BMI > 95th	4
Momeni [42]	2020/2016	Cross-sectional	Kerman/Kerman	5–6	731 (376/355)	9.1%, 8%	BMI: 85th–95th, BMI > 95th	4
Eslami [43]	2020/2018	Cross-sectional	Tehran/Tehran	7–10	356 (191/165)	21.6%, 13.5%	BMI: 85th–95th, BMI > 95th	4
Golpour-Hamedani [44]	2020/NM	Cross-sectional	Isfahan/Isfahan	11–18	456 (189/267)	17.1%, 9%	BMI: 85th–95th, BMI > 95th	4
Mousapour [45]	2020/1999–2017	Cohort	Tehran/Tehran	> 19	10,045 (4480/5565)	4.5%	BMI > 35	5
Mirzaei [46]	2020/2011	Cross-sectional	Yazd/Yazd	20–69	8749 (4349/4400)	Obesity: 26.3%	BMI > 30.0	5
Kheradmand [47]	2019/2015–2017	Cross-sectional	Mazandaran/Sari	35–70	10,255 (4149/6106)	42.4%, 33.5%	BMI 25.0–29.9/BMI > 30.0	5
Emamian [48]	2019/2015	Cross-sectional	Semnan/Shahroud	9.7 ± 1.7	5586 (3011/2575)	15.1%, 9.6%	1SD < BMI < 2SD/BMI > 2SD	5
Abiri [49]	2019/2017–2018	Cross-sectional	Tehran/Tehran	14–17	2132 females	24.1%, 6.5%	BMI: 85th–95th, BMI > 95th	5
Eidkhani [50]	2019/phase 1: 1999–2005/phase 2: 2012–2014	Cohort	Tehran/Tehran	11–19	Phase 1: 2998 (1415/1583); Phase 2: 946 (477/496)	Phase 1: 10.9%, 13.3% Phase 2: 15.1%, 2.4%	BMI: 85th–95th, BMI > 95th	6
Momeni [51]	2019/2017	Cross-sectional	Kerman/Kerman	40–60	450 (225/225)	42.8%, 21.3%	BMI 25.0–29.9/BMI > 30.0	4
Yousefichaijan [52]	2019/NM	Cross-sectional	Markazi/Arak	≥ 5	994 (508/486)	13.2%, 10.5%	BMI: 85th–95th, BMI > 95th	4
Khodabakhshi [53]	2019/2014	Cross-sectional	Khorasn Jonobi/Birjand	60–86	400 (200/200)	49%, NM	BMI > 25.0	4
Jalali-Farahani [54]	2019/NM	Cross-sectional	Isfahan/Isfahan	12–18	584 (276/308)	18.5%, 11.8%	1SD < BMI < 2SD/BMI > 2SD	4
Hajian-Tilaki [55]	2019/NM	Cross-sectional	Mazandaran/Babol	42.8 ± 13.4	981 (443/538)	Overweight and obesity: 67.1%	BMI > 25.0	4
Karimi [56]	2019/2017	Cross-sectional	Markazi/Zarandieh	6.3 ± 1.1	572 (287/285)	15.5%, 9.9%	BMI 25.0–29.9/BMI > 30.0	4
Zohal [57]	2019/2014–2017	Cross-sectional	Yazd	42.26 ± 13.03	149 (48/101)	44.3%, 30.9%	BMI 25.0–29.9/BMI > 30.0	4
Mohammadi [58]	2019/2017	Cross-sectional	Chaharmahal and Bakhtiari/Farsan	6–12	1189 (515/674)	Males: 9.9%, 8.5% Females: 8.6%, 3.4%	BMI: 85th–95th, BMI > 95th	4
Karimy [56]	2019/2017	Cross-sectional	Markazi/Zarandieh	6–7	432 (214, 218)	Obesity: 12.2.%	BMI > 95th	3
Pouraram [59]	2018/2011–2015	Cross-sectional	All 31 provinces of Iran	14–60	32,770 (both sexes, not reported their numbers)	Adolescents: 15.9%, 6.9%	BMI: 85th–95th, BMI > 95th	5
Rezaei [60]	2018/2014–2016	Cross-sectional	Kermanshah/Ravansar	35–65	8822 (4199/4623)	43.4%, 27.6%	BMI 25.0–29.9/BMI > 30.0	5
Najafi [61]	2018/2014–2016	Cross-sectional	Kermanshah/Ravansar	35–65	10,086 (4786/5300)	26.7%	BMI > 30.0	6
Gandomkar [62]	2018/2012–2014	Cross-sectional	Fars/Valashahr	52.6 ± 9.7	9264 (4276/4988)	37.3%, 18.2%	BMI 25.0–29.9/BMI > 30.0	6
Ejtahed [63]	2018/2015	Cross-sectional	30 provinces of Iran	7–18	14,002 (7085/6917)	9.4%, 11.4%	BMI: 85th–95th, BMI > 95th	6
Tabrizi [64]	2018/2015	Cross-sectional	East Azerbaijan	15–65	2818 (1368/1450)	39.6%, 24%	BMI 25.0–29.9/BMI > 30.0	6
Ghaderian [65]	2018/2015	Cross-sectional	Khuzestan/Ahvaz	41.07 ± 13.52	2575 (1187/1388)	38.9%, 26.5%	BMI 25.0–29.9/BMI > 30.0	6
Kolahi [66]	2018/2011	Cross-sectional	All 31 provinces of Iran	> 20	12,000 (3879/5618)	34.5%, 21.5%	BMI 25.0–29.9/BMI > 30.0	5
Motlagh [67]	2018/2015–2016	Cross-sectional	Five ethnicities in the Islamic Republic of Iran: Arab, Kurdish, Sistani and Baluchi, Turkish and Turkmen	12–14	2444 (1271/1173)	15.3%, 9.2%	BMI: 85th–95th, BMI > 95th	5
Molani Gol [68]	2018/2017	Cross-sectional	East Azerbaijan/Tabriz	30–55	152 males	34.2%, 52.6%	BMI 25.0–29.9/BMI > 30.0	4
Rashidi [69]	2018/phase 1: 2009; phase 2: 2014	Cohort	Khouzestan/Ahvaz	10–16	176 (100/76)	Phase 1: 7.4%, 14.8% Phase 2: 10%, 4%	BMI: 85th–95th, BMI > 95th	4
Mohammad Beigi [70]	2018/2015	Cross-sectional	Qom/Qom	21 ± 2	301 (187/113)	21.3%	BMI > 30.0	4
Ardekani [71]	2018/2014–2016	Cross-sectional	Yazd/Yazd	20–70	8652 (4291/4361)	Obesity: 25.9%	BMI > 30.0	5
Maleki [72]	2019/NM	Cross-sectional	Lorestan/borujerd	≥ 35	801 (412, 389)	Obesity: 22.8%	BMI > 30.0	5
Ghanbari Ghozikali [73]	2017/2016	Cross-sectional	East Azerbaijan/Tabriz	15–18	1143 males	18.5%, 10.1%	BMI: 85th–95th, BMI > 95th	4
Kelishadi [74]	2017/2011–2012	Cross-sectional	30 provinces of Iran	6–18	36,529 (18,553/17,976)	11.5%, 8.3%	BMI: 85th–95th, BMI > 95th	6
Djalalinia [75]	2017/NM	Cross-sectional	30 provinces of Iran	6–18	21,876 (11,132/10,744)	13.1%, 6.5%	BMI: 85th–95th, BMI > 95th	6
Amani [76]	2017/2015	Cross-sectional	Kurdistan/7 cities	≥ 20	13,710 (both sexes, not reported their numbers)	19.3%	BMI > 25.0	6
Emamian [48]	2019/2005	Cross-sectional	31 provinces in Iran	15–64	87,240 (43,773, 43,467)	13.1%	BMI > 30.0	7
Jahromi [77]	2017/2014	Cross-sectional	Fars/Jahrom	31.04 ± 6.97	263 (53, 210)	Overweight: 19.3%	BMI: 25.0–30	4
Raeisi [78]	2017/NM	Cohort	Bushehr	67.9 ± 7.1	3000 (1455/1545)	Males: 57.7%, 14.7% Females: 73.2%, 33.7%	BMI 25.0–29.9/BMI > 30.0	5
Saber [79]	2017/2010	Cross-sectional	Kerman/Kerman	15–75	988 (420/568)	36.6%, 18.1%	BMI 25.0–29.9/BMI > 30.0	4
Hajian-Tilaki [80]	2017/2012	Cross-sectional	Mazandaran/Babol	20–70	986 (446/540)	38%, 29.1%	BMI 25.0–29.9/BMI > 30.0	4
Mirshekar [81]	2017/2016	Cross-sectional	Sistan and Baluchestan/Zabol	7–11	3443 (1748/1695)	10.9%, 4.5%	BMI: 85th–95th, BMI > 95th	5
Abbasalizad Farhangi [82]	2017/NM	Cross-sectional	East Azarbayjan	60–94	248 (110/138)	50%	BMI > 25.0	4
Ghobadi [83]	2017/2015–2016	Cross-sectional	Fars/Shiraz	6–10	607 (276/331)	9.1%, 8.4%	BMI: 85th–95th, BMI > 95th	4
Doostan [84]	2017/2012	Cross-sectional	Kerman/Kerman	2–6	1154 (597/557)	2.4%, 4.5%	1SD < BMI < 2SD/BMI > 2SD	4
Nikooyeh [85]	2017/2013	Cross-sectional	Six provinces of Iran	5–18	667 (322/345)	6.7%, 4.1%	BMI: 85th–95th, BMI > 95th	4
Doustmohamadian [86]	2016/1999	Cross-sectional	Tehran/Tehran	≥ 30	8804 (3974/4830)	70.9%, 26.6%	BMI > 25.0/BMI > 30.0	5
Gandomkar [87]	2016/2012–2014	Cross-sectional	Fars/Valashahr	40–75	9264 (4276/4988)	37.3%, 18.1%	BMI 25.0–29.9/BMI > 30.0	6
Javedan [88]	2016/2015	Cross-sectional	Tehran/Tehran	5–7	17,487 (8888/8599)	19.9%, 6.8%	BMI: 85th–95th, BMI > 95th	6
Jalali-Farahani [89]	2016/2008–2010	Cross-sectional	Tehran/Tehran	≥ 20	2747 (1146/1601)	61.6%	BMI > 25.0	5
Hassanzadeh-Rostami [90]	2016/2012–2013	Cross-sectional	Fars/Shiraz	2–6.9	8821 (4618/4203)	5.7%, 5.2%	BMI: 85th–95th, BMI > 95th	6
Badeli [91]	2016/2013–2015	Cross-sectional	Guilan/Rasht	7–17	2062 (1153/909)	13.3%, 3.5%	BMI 25.0–29.9/BMI > 30.0	5
Zarrati [92]	2016/2011–2012	Cross-sectional	Tehran/Tehran	11–14	1184 (559, 625)	22%, 5.3%	BMI: 85th–95th, BMI > 95th	5
Veghari [93]	2016/2013	Cross-sectional	Golestan	≤ 5	2487 (1280/1207)	5.2%, 3.5%	1SD < BMI < 2SD/BMI > 2SD	5
Nikniaz [94]	2016/2013	Cross-sectional	East Azerbaijan/Tabriz	18–65	500 females	20.2%, 24.6%	BMI 25.0–29.9/BMI > 30.0	4
Keykhaei [95]	2016/2012	Cross-sectional	Sistan and Baluchistan/Zahedan	7–11	585 (293/292)	11.7%, 22%	BMI: 85th–95th, BMI > 95th	4
Bakhshi [96]	2016/2000–2011	Cohort	All 31 provinces of Iran	> 20	2000: 27,869 (12,690/15,179); 2007: 26,716 (13,194/13,522); 2009: 20,917 (10,083/10,834); 2011: 8425 (3551/4874)	2000: 12.3%; 2007: 20.7%; 2009: 20.2%; 2011: 22.2%	BMI > 30.0	6
Bahrani [97]	2016/NM	Cross-sectional	Fars/Shiraz	16.3 ± 1.0	538 (289/249)	18%, 6.7%	1SD < BMI < 2SD/BMI > 2SD	4
Pourghasem [98]	2016/2016	Cross-sectional	Mazandaran/Babol	6–18	1158 (653/505)	8%, 2.2%	BMI 25.0–29.9/BMI > 30.0	4
Baygi [99]	2016/2015	Cross-sectional	Iranian male seafarers of NITC	36.0 ± 10.3	234 males	42.5%, 8.6%	BMI 25.0–29.9/BMI > 30.0	4
Jamalikandazi [100]	2016/NM	Cross-sectional	Ilam/Ilam	16–18	360 females	10.8%, 5%	BMI: 85th–95th, BMI > 95th	4
Heidari-Bakavoli [101]	2015/started in 2010	Cohort	Khorasan/Mashhad	35–65	9765 (3907/5866)	42.8%, 30.9%	BMI 25.0–29.9/BMI > 30.0	6
Hovsepian [102]	2015/2011–2012	Cross-sectional	30 provinces of Iran	6–18	13,486 (6846/6640)	9.6%, 11.8%	BMI: 85th–95th, BMI > 95th	6
Esmaili [103]	2015/2011–2012	Cross-sectional	30 provinces of Iran	6–18	13,486 (6943/6543)	9.6%, 11.8%	BMI: 85th–95th, BMI > 95th	6
Kelishadi [104]	2015/2011–2012	Cross-sectional	31 provinces of Iran	6–18	23,043 (11,706/11,337)	13%, 6.6%	BMI: 85th–95th, BMI > 95th	7
Ghadimi [105]	2015/2012	Cross-sectional	Mazandaran/Babol	7–11	3649 (1780/1869)	11.7%, 14.3%	BMI: 85th–95th, BMI > 95th	6
Heshmat [106]	2015/2009–2010	Cross-sectional	27 provinces of Iran	10–18	5570 (2786/2784)	80%, 8.7%	BMI: 85th–95th, BMI > 95th	6
Soheilipour [107]	2015/2012	Cross-sectional	Sistan and Baluchestan/Zahedan	6–13	3582 (1796/1796)	11.8%, 92.9%	BMI: 85th–95th, BMI > 95th	5
Kalani [108]	2015/NM	Cross-sectional	Yazd/Yazd	≥ 18	1130 (456/674)	36.1%, 26.1%	BMI 25.0–29.9/BMI > 30.0	4
Abdollahi [109]	2015/2008	Cross-sectional	Tehran/Pakdasht	NM	1178 females	37.3%, 20.3%	BMI 25.0–29.9/BMI > 30.0	4
Shojaei [110]	2015/2015	Cross-sectional	Fars/Jahrom	> 30	892 (405/487)	85.7%, 34.7%	BMI 25.0–29.9/BMI > 30.0	4
Barzin [111]	2015/1999–2011	Cohort	Tehran/Tehran	≥ 20	1999–2001: 10,368 (4397/5971); 2009–2011: 6217 (2573/3644)	1999–2001: 23.1%; 2009–2011: 34.1%	BMI > 30.0	5
Bagheri Lankarani [112]	2015/2010–2011	Cross-sectional	Fars/Shiraz	18–88	777 (326/451)	39.6%, 18.7%	BMI 25.0–29.9/BMI > 30.0	4
Vaziri-Esfarjani [113]	2015/2009	Cross-sectional	Khouzestan/Ahvaz	7–11	960 (480/480)	11.9%, 6%	BMI: 85th–95th, BMI > 95th	4
Alimoradi [114]	2015/NM	Cross-sectional	Qazvin/Qazvin	10–18	318 (162, 156)	15.7%, 4.7%	BMI: 85th–95th, BMI > 95th	4
Ghiyas Tabari [115]	2015/2015	Cross-sectional	Sistan and Baluchestan/Chabahar	28.310 ± 9.11	120 females	15%, 7.5%	BMI 25.0–29.9/BMI > 30.0	4
Kelishadi [116]	2015/NM	Cross-sectional	27 provinces in Iran	10–18	5625 (2824/2801)	80%, 8.9%	BMI: 85th–95th, BMI > 95th	5
Kelishadi [117]	2014/2009	Cross-sectional	31 provinces of Iran	6	955,388 (492,025/463,363)	10.9%, 3.4%	BMI: 85th–95th, BMI > 95th	7
Heidari [118]	2014/2010	Cross-sectional	Isfahan/Isfahan	12–14	12,946 (2415/10,531)	17.1%, 22.1%	BMI: 85th–95th, BMI > 95th	6
Tabesh [119]	2014/2012–2013	Cross-sectional	Khuzestan/Ahvaz	7–11	5811 (2904/2907)	Males: 23.6%, 6.05% Females: 19.3%, 4.5%	BMI: 85th–95th, BMI > 95th	5
Ataie-Jafari [120]	2014/2009–2010	Cross-sectional	27 provinces of Iran	10–18	4641 (2326/2315)	10.9%, 11.8%	BMI: 85th–95th, BMI > 95th	5
Rahmanian [121]	2014/2009–2010	Cross-sectional	27 provinces of Iran	10–18	5088 (2556/2532)	9.1%, 7.55	BMI: 85th–95th, BMI > 95th	5
Jafari [122]	2014/2009–2010	Cross-sectional	Isfahan/Isfahan	9–15	4691 (2347/2344)	10.4%, 5.7%	BMI: 85th–95th, BMI > 95th	5
Shafaghi [123]	2014/2010–2011	Cross-sectional	Khorasan Razavi/Mashhad	13.1 ± 1.03	1189 (579/610)	17.2%, 11.9%	1SD < BMI < 2SD/BMI > 2SD	5
Hatami [124]	2014/2009–2010	Cross-sectional	Tehran/Tehran	10–18	1157 (NM)	Overweight and obesity: 19.8%	BMI ≥ 1 SD	4
Jalali-Farahani [125]	2014/NM	Cross-sectional	Tehran/Tehran	14–17	465 (238/227)	Overweight and obesity: 38.4%	BMI ≥ 1 SD	4
Heidari-Beni [126]	2014/2013	Cross-sectional	Isfahan/Isfahan	11–13	205 females	Overweight and obesity: 50.7%	BMI ≥ 1 SD	4
Nouri Saeidlou [127]	2014/2011	Cross-sectional	West Azerbaijan, Kermanshah, Isfahan	< 5	West Azerbaijan: 902 (447, 455); Kermanshah: 829 (406, 423); Isfahan: 794 (400, 394)	West Azerbaijan: 5.1%, 1.3%; Kermanshah: 4.5%, 0.7%; Isfahan: 3.6%, 0.1%	1SD < BMI < 2SD/BMI > 2SD	5
Farzaneh [128]	2014/2013	Cross-sectional	Mazandaran/Amol	14–15	381 (148, 233)	16.6%, 6.6%	BMI 25.0–29.9/BMI > 30.0	4
Asgari [129]	2013/2009	Cross-sectional	All 31 provinces of Iran	≥ 20	20,917 (10,083/10,834)	20.2%	BMI > 30.0	6
Yarahmadi [130]	2013/2010–2011	Cross-sectional	6 provinces' capital cities (Isfahan, Karaj, Mashad, Shiraz, Tabriz, and Tehran)	≥ 30	439,406 (175,762, 263,644)	27%, 20%	BMI 25.0–29.9/BMI > 30.0	7
Naderi Beni [131]	2013/2010	Cross-sectional	Isfahan/Chadegan	≤ 5	403 (NM)	2.2%	BMI > 2 SD	4
Veghari [132]	2013/2010	Cross-sectional	Golestan	15–65	2994 (1499/1495)	31.7%, 22.7%	BMI 25.0–29.9/BMI > 30.0	5
Khashayar [133]	2013/2009–2010	Cross-sectional	All 31 provinces of Iran	10–18	5738 (2863/2875)	Overweight and obesity: 17.7%	BMI ≥ 1 SD	5
Basiratnia [134]	2013/2010–2011	Cross-sectional	Fars/Shiraz	13.85 ± 1.69	2000 (953, 1047)	13%, 7%	BMI: 85th–95th, BMI > 95th	5
Ghavamzadeh [135]	2013/2008–2009	Cross-sectional	West Azerbaijan/Urmia	11–20	2498 (NM)	Overweight and obesity: 14.1%	BMI ≥ 1 SD	5
Damirchi [136]	2013/2012	Cross-sectional	Guilan/Rasht	18–69	400 (200, 200)	52%, 23.5%	BMI 25.0–29.9/BMI > 30.0	4
Koushki [137]	2013/2012	Cross-sectional	Khorasan Razavi/Neyshabur	30–50	381 females	45.5%, 30.4%	BMI 25.0–29.9/BMI > 30.0	4
Taheri [138]	2013/2012	Cross-sectional	South Khorasan/Birjand	6–11	1541 (690, 851)	9.5%, 9.2%	BMI: 85th–95th, BMI > 95th	5
Khoshandam Sarvynehbaghi [139]	2013/2010	Cross-sectional	Mazandaran/NM	20–66	400 males	47.2%, 24.8%	BMI 25.0–29.9/BMI > 30.0	4
Bahreini [140]	2013/2010	Cross-sectional	Isfahan/Isfahan	11–18	3002 (1377/1652)	5.6%, 3.5%	BMI: 85th–95th, BMI > 95th	5
Dalili [141]	2013/NM	Cross-sectional	Guilan/Rasht	12	752 (493, 259)	15%, 16.5%	BMI: 85th–95th, BMI > 95th	4
Hajian-Tilaki [142]	2013/2012	Cross-sectional	Mazandaran/Babol	2–5	760 (375, 385)	11.8%, 15%	BMI: 85th–95th, BMI > 95th	5
Hatami [143]	2013/2009–2010	Cross-sectional	Tehran/Tehran	12–18	739 (339/400)	13.9%, 6.6%	1SD < BMI < 2SD/BMI > 2SD	4
Salehi-Abargouei [144]	2013/NM	Cross-sectional	Isfahan/Isfahan	6–12	635 (159, 476)	Obesity: 17.6%	BMI > 95th	4
Loukzadeh [145]	2013/2012	Cross-sectional	Yazd/NM	36 ± 8.8	152 males	48%, 15.1%	BMI 25.0–29.9/BMI > 30.0	4
Agha-Alinejad [146]	2013/NM	Cross-sectional	Tehran/Tehran	5–6	381	7.1%, 12%	BMI: 85th–95th, BMI > 95th	4
Jafari [147]	2012/NM	Cross-sectional	Golestan/NM	40–75	50,045 (21,219, 28,826)	33.9%, 25.4%	BMI 25.0–29.9/BMI > 30.0	6
Mohebbi [148]	2012/2007–2010	Cross-sectional	Professional long-distance drivers	20–67	12,138 males	41.4%, 21.3%	BMI 25.0–29.9/BMI > 30.0	6
Poorolajal [149]	2012/2005–2009	Cross-sectional	Hamadan/Hamadan	15–64	6500 (3250, 3250)	32.1%, 15.1%	BMI 25.0–29.9/BMI > 30.0	6
Barzigar [150]	2012/2009	Cross-sectional	Guilan/14 cities	15–89	2283 (1062, 1221)	65.5%	BMI > 25.0	6
Heidari [151]	2012/2006–2008	Cross-sectional	Tehran/Tehran	41.0 ± 7	1041 females	45.8%, 35.7%	BMI 25.0–29.9/BMI > 30.0	5
Doustmohammadian [152]	2012/2001–2003	Cross-sectional	31 provinces of Iran	11–19	7908 (4158, 3750)	7.3%, 3.3%	BMI: 85th–95th, BMI > 95th	6
Shirani [153]	2012/2007	Cross-sectional	Isfahan/Isfahan	15–64	1000 (500, 500)	60.8%	BMI > 25.0	5
Gharakhanlou [154]	2012/NM	Cross-sectional	Tabriz/7 big cities	15–74	2179 (991, 1188)	Males: 39.4%, 10.2% Females: 34.4%, 18.6%	BMI > 25.0/BMI > 30.0	6
Hajian-Tilaki [155]	2012/2008	Cross-sectional	Mazandaran/Babol	12–17	1199 (600/599)	15%, 8.3%	BMI: 85th–95th, BMI > 95th	5
Esmaeilzadeh [156]	2012/NM	Cross-sectional	Ardabil/Ardabil	7–11	766 males	14.1%, 4.1%	BMI 25.0–29.9/BMI > 30.0	4
Vafa [157]	2012/2008	Cross-sectional	Tehran/Tehran	7	511 (235, 276)	8%, 11.7%	BMI: 85th–95th, BMI > 95th	4
Mirhosseini [158]	2012/2007	Cross-sectional	Khorasan Razavi/Mashhad	15–18	477 females	14.6%, 3.4%	1SD < BMI < 2SD/BMI > 2SD	4
Mehrkash [159]	2012/NM	Cross-sectional	Golestan/	15–18	450 (225/225)	Overweight: 10.5%	BMI: 85th–95th	4
Najafipour [160]	2012/2009–2011	Cross-sectional	Kerman/Kerman	15–75	5900 (2662/3238)	Obesity: 17.7%	BMI > 30.0	5

M/F, male/female; NOS, Newcastle–Ottawa Scale; F/M, female/male; BMI, body mass index; NM, not mentioned; NITC, National Iranian Tanker Company

## Statistical analysis

Statistical analyses were performed using Stata, version 17.0 (Stata Crop, College Station, TX, USA). Random effect models were used to estimate the prevalence of overweight and obesity. Heterogeneity among the studies was evaluated using *I*^2^ and Q2 statistics. Subgroup analyses were performed by age group (≤ 18 or > 18 years) to identify any alteration in the results of the study. We used meta-regression to evaluate the relationship between the year of data collection and year of publication, and the prevalence of overweight and obesity.

## Results

By conducting a primary search of the keywords associated with our topic, we found 3176 full-text articles. Among them, 1982 articles were obtained from English databases, and the rest were from Persian databases. After excluding overlapping studies and considering the inclusion and exclusion criteria in two separate steps (title and abstract review), we selected 152 (74 for ≤ 18 years, 61 for > 18 years, and 17 for combination of age groups) qualified studies for inclusion in our review. Figure 1 provides a summary of the primary research results and the process of selecting the appropriate studies.

This meta-analysis included 152 articles and 2,456,489 participants in total [11–160] (Table 1). The sample size of these articles varied between 120 participants and 955,388 and more than 1000 people were included in 61.8% of the studies. The prevalence of obesity in children and adolescents (age ≤ 18 years) was reported in 74 articles (48.7%), whereas in 61 articles (40.1%), the age of the study participants was > 18 years. A combination of these age groups was reported in 17 studies. Generally, the excess weight (overweight and obesity) reported in articles ranges from 1.4 to 92.9%. Khouzestan (Ahvaz) and Fars (Jahrom) had the lowest and highest rates of overweight/obesity, respectively. When it comes to individuals aged 18 or younger, West Azerbaijan and Zahedan are the regions of concern with the lowest and highest prevalence, respectively.

Based on reports published between 2012 and 2021, the percentage of overweight and obese individuals in Iran was estimated at 20.1 (95% CI 17.92–22.30) and 13.44 (95% CI 11.76–15.22), respectively. This percentage (95% CI) was 11.71 (10.98–12.46) for overweight and 8.08 (7.02–9.22) for obesity in those aged ≤ 18 years, and 35.26 (32.61–37.99) for overweight and 21.38 (19.61–23.20) for obesity in those aged > 18 years (Table 2). At the same time, the overall prevalence of overweight and obesity in the entire population was 35.09% (95% CI 31.31–38.98) (Table 2).

**Table 2:** The percentage of individuals with overweight and/or obesity among Iranian population

Outcome	Overall/subgroups	Number of effect size	Pooled ES (95%CI)	*I*^2^	*P* heterogeneity
Prevalence of overweight and obesity	Overall	132	35.09 (31.31,38.98)	99.69	< 0.01
	≤ 18 years	93	21.11 (19.56,22.70)	99.52	< 0.01
	> 18 years	67	56.55 (52.67,60.38)	99.91	< 0.01
Prevalence of obesity	Overall	166	13.44 (11.76,15.22)	99.92	< 0.01
	≤ 18 years	91	8.08 (7.02,9.22)	99.57	< 0.01
	> 18 years	75	21.38 (19.61,23.20)	99.79	< 0.01
Prevalence of overweight	Overall	146	20.10 (17.98,22.30)	99.92	< 0.01
	≤ 18 years	87	11.71 (10.98,12.46)	98.58	< 0.01
	> 18 years	59	35.26 (32.61,37.99)	99.82	< 0.01

ES, effect size; CI, confidence interval

**Fig. 2:**
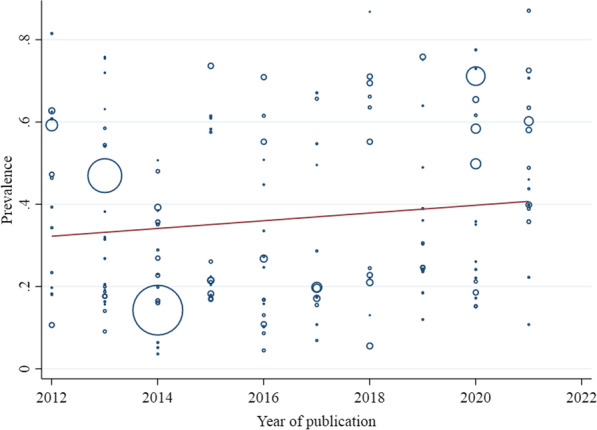
Association between year of publication and prevalence of overweight in all age groups

**Fig. 3:**
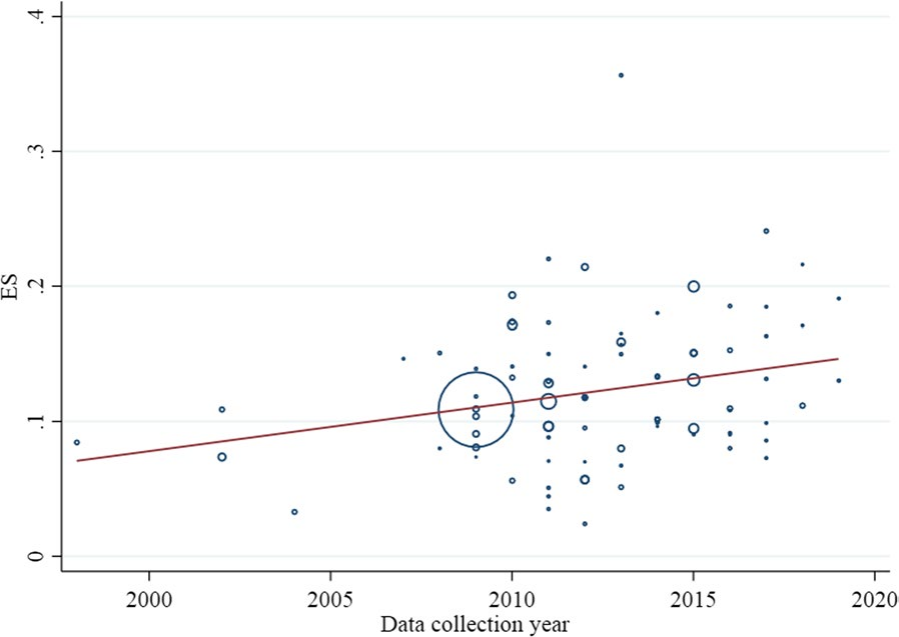
Association between year of data collection and prevalence of overweight in individuals aged ≤ 18 years

**Fig. 4:**
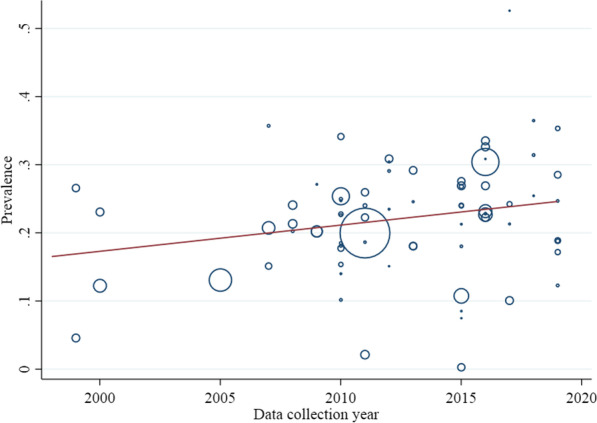
Association between year of data collection and prevalence of obesity in adults

In meta-regression models, from the year of data collection and year of publication, there was a significant association between the year of publication and the prevalence of overweight among all ages (coefficient = − 0.008, SE = 0.004, *P* value = 0.04) (Fig. 2). According to the model, a statistically significant association was found between the year of data collection and overweight prevalence in ≤ 18 years (coefficient = − 0.003, SE = 0.001, *P* value = 0.01) (Fig. 3) and prevalence of obesity in the adult population (coefficient = − 0.003, SE = 0.001, *P* value = 0.04) (Fig. 4). However, in other cases, meta-regression did not show any association between the prevalence of overweight and/or obesity and year of data collection or publication (Table 3).

**Table 3:** Meta-regression for the effects of year of data collection and publication

Variable	Potential confounder	Coefficient	SE	*P* value
*All age groups*				
Prevalence of overweight	Year of data collection	0.005	0.003	0.07
	Year of publication	0.008	0.004	**0.04**
Prevalence of obesity	Year of data collection	0.002	0.001	0.14
	Year of publication	0.004	0.002	0.11
Prevalence of overweight and obesity	Year of data collection	0.006	0.004	0.14
	Year of publication	0.009	0.005	0.10
≤ *18 years*				
Prevalence of overweight	Year of data collection	0.003	0.001	**0.01**
	Year of publication	0.001	0.002	0.45
Prevalence of obesity	Year of data collection	0.0005	0.001	0.72
	Year of publication	− 0.001	0.002	0.66
Prevalence of overweight and obesity	Year of data collection	0.005	0.003	0.04
	Year of publication	0.003	0.004	0.41
> *18 years*				
Prevalence of overweight	Year of data collection	− 0.000009	0.003	0.99
	Year of publication	0.0006	0.004	0.88
Prevalence of obesity	Year of data collection	0.003	0.001	**0.04**
	Year of publication	0.001	0.003	0.61
Prevalence of overweight and obesity	Year of data collection	− 0.002	0.005	0.62
	Year of publication	− 0.0007	0.006	0.90

SE, standard error

Values in bold indicates P < 0.05

**Fig. 5:**
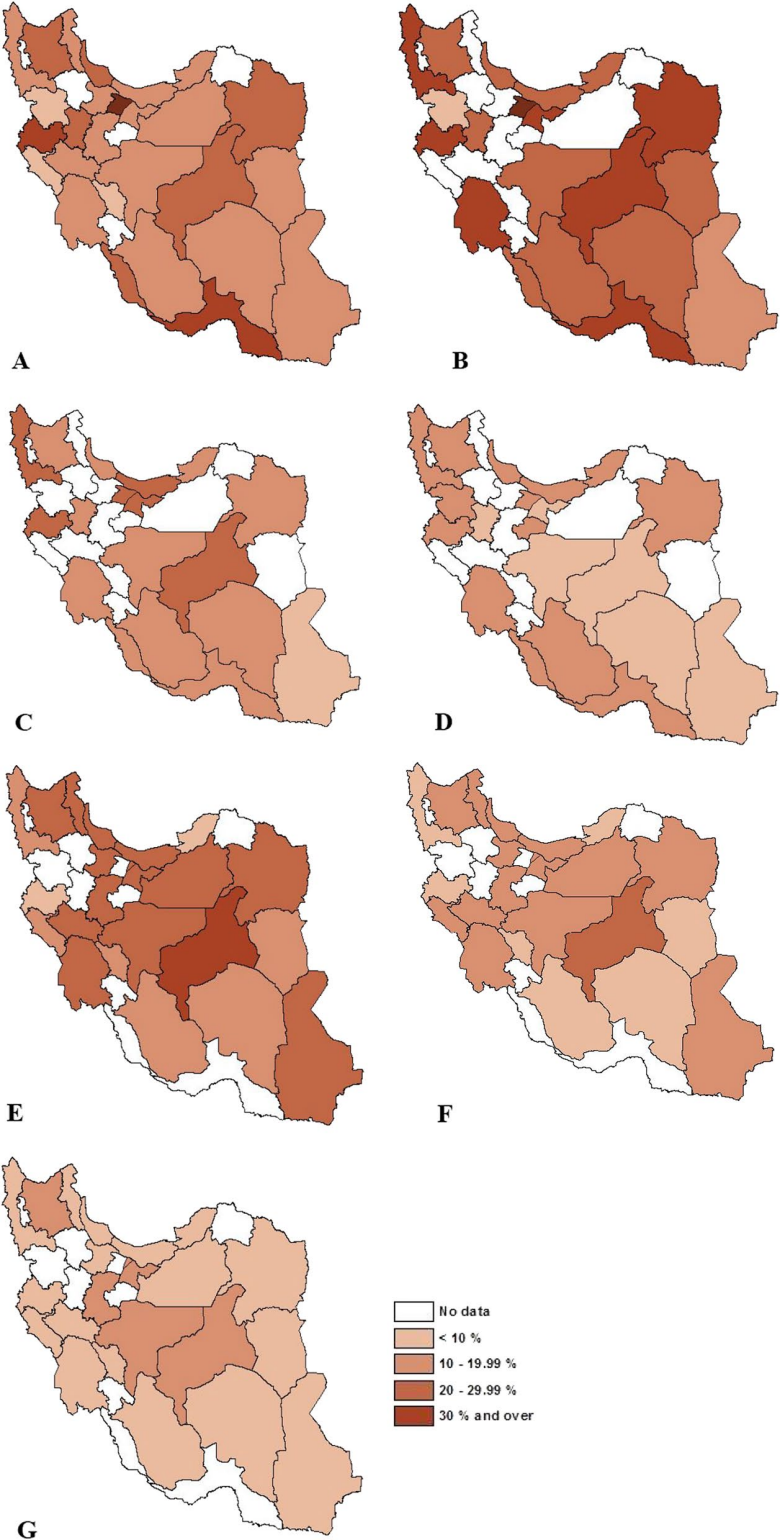
**A** Geographical dispersion of overweight and obesity in all age groups across the country. **B** Geographical dispersion of overweight and obesity in adults. **C** Geographical dispersion of overweight in adults. **D** Geographical dispersion of obesity in adults. **E** Geographical dispersion of overweight and obesity in aged ≤ 18 years. **F** Geographical dispersion of overweight in aged ≤ 18 years. **G** Geographical dispersion of obesity in aged ≤ 18 years

According to the published reports between 2012 and 2021, as a whole, the highest prevalence of overweight and obesity was observed in Alborz, Kermanshah, Hormozgan, Bushehr, East Azerbaijan, Yazd, Hamedan, and Guilan provinces, respectively. Throughout the years, these numbers were the lowest in Lorestan, Kordistan, Ilam, and Chaharmahal va Bakhtiari (Fig. 5A). The distribution of the prevalence of overweight and/or obesity by age group is shown in Fig. 5B–G.

## Discussion

Based on published data from 2012 to 2021, we estimated the prevalence of overweight and obesity in the Iranian population according to age and geographical distribution. Accordingly, the total rate of overweight and obesity in Iran was estimated to be 35.09%, which was calculated to be 56.55% for people older than 18 years and 21.11% for people younger than 18 years.

Since obesity prevalence is on the rise in Iran, as well as the health and socioeconomic problems resulting from it, it is crucial to study obesity and access comprehensive and precise data to assess public health and to determine relevant health policies and obesity prevention measures. Hence, the World Health Organization continues to encourage countries to use the burden of disease to guide policymaking at the national level [161].

In the latest meta-analysis on overweight and obesity prevalence in Iran [161], which was carried out according to published data between 1995 and 2011, the prevalence of obesity in populations above the age of 18 was estimated to be 21.7% (CI 95% 18.5–25%) and in populations below 18, 6.1% (CI 95% 6.8–5.4%). In another pooled analysis [162] between the adult population aged 25 to 85 years, during 1990–2016, the prevalence rates of overweight and obesity were 71.7% (67.9–75.8) and 36.8% (34.1–39.7) in females and 57.1% (53.7–60.6) and 18.4% (16.9–20) in men, respectively. A meta-analysis [2] estimated that in 2014, the prevalence of obesity and overweight among Iranian children and adolescents was approximately 5.1% (95% CI 4.4–5.8) and 10.8% (95% CI 10.2–11.4). An analysis of obesity data until 2005 conducted by Mirzazadeh estimated the obesity rate among people over 18 to be 21.5% and the rate among those under 18 to be 5.5% [163]. The difference between the current study and previous studies was due to the difference in the time of conducting the studies, extent of the data, and larger sample size.

Obesity is the most obvious indication of an inappropriate sedentary lifestyle and increase in high-calorie food consumption [164]. Based on a study published in 2005, approximately 937 million adults (23.2%) were estimated to be overweight, whereas approximately 396 million adults (24%) were estimated to be obese. According to projections, there will be 1.35 billion overweight and obese people and 573 million obese people by 2030 [165]. According to the National Health and Nutrition Examination Survey in 2010, more than one-third of the adults in the USA were obese [166]. In another study of the adult population in Spain, the prevalence of obesity was 22.9% (24.4% in men and 21.4% in women) [167]. Turkey had an overweight prevalence of 19.0% %in 2007 (17.4% in men and 20.4% in women) [168]. The prevalence of obesity in Asian countries is 10.3% and that of overweight and obesity is 25.0% in Pakistan [169]. Overweight and obesity were more prevalent in our review than in the other studies.

The difference in obesity prevalence rates worldwide is influenced by various factors, such as age, sex, race, ethnicity, lifestyle, and socioeconomic status. Additionally, these factors have a significant impact on obesity prevalence [161].

Furthermore, obesity/overweight prevalence varies widely across provinces in Iran, likely due to different cultural affiliations and socioeconomic factors. The multiethnic nature of Iran directly affects eating habits and lifestyles in every region due to cultural, environmental, and genetic variations. Environmental factors such as climate variability affect diet, mood, and activity levels. As a result of urbanization, obesity is noticeably increasing in Iran and is considered a contributing factor [1].

In addition, over the last 3 decades, obesity among children and adolescents has more than doubled [170]. In 2010, the National Center for Health Statistics (NCHS) reported that almost 17% of the youth were obese [166]. Childhood obesity increases the likelihood of developing obesity in adults. Increasing obesity at a young age increases the risk of obesity-related diseases in children, including diseases that were once called adult diseases, such as type-2 diabetes. This issue requires further investigation. In 1980, obesity rates among children and adolescents in the USA were 7% and 5%, respectively, whereas in 2012 they reached nearly 18% and 21%, respectively [171]. In 2005, the percentage of overweight or obese children and adolescents in South Korea was 9.7% (11.3% boys and 8.0% girls) [172]. According to the MONICA project (monitoring of cardiovascular diseases) in 1998, Iran is one of the seven countries with high childhood obesity rates [173]. Even a small increase in BMI in children may result in long-term health effects [2].

Like other developing countries, Iran has experienced an increase in BMI primarily as a result of lifestyle changes that have occurred in recent decades due to rapid socioeconomic development, westernized lifestyles, inadequate physical activity, urbanization, and urban–rural migration [162]. However, there is a complex combination of biological and non-biological factors that can influence lifestyle habits such as age, sex, race, and socioeconomic background [174, 175]. Iran has a higher prevalence of obesity than most countries in Middle East-North Africa (MENA), including Afghanistan, Turkmenistan, and Pakistan [162]. Considering the clinical significance of the underlying pathophysiology, which is mainly shared among populations and can be explained through experimental and clinical studies, it is important to investigate the distribution of increased BMI in Iran.

In this study, we conducted a comprehensive systematic search of all published sources of information on the prevalence of overweight and obesity in the Iranian population between 2012 and 2021. All domestic databases were searched using English and Persian equivalent terms. However, this study has some limitations. It is difficult to compare data between studies because of the differences in the groups studied, differences in living areas, and discrepancies in measures. Another limitation of this meta-analysis is that it did not report the prevalence of overweight/obesity based on urban or rural areas, due to data limitations, and did not investigate the relationship between obesity and socioeconomic status. In addition, the results of our meta-analysis may not be applicable to the general population, because the prevalence data for overweight and obesity in the included studies were not exclusively based on healthy individuals.

Further research should be conducted to investigate the influence of sex, age, health status, rural and urban areas, and socioeconomic factors on the obesity prevalence.

## Conclusion

Considering past meta-analyses, the present study concludes that overweight/obesity is on the rise among Iranians of all ages and sexes. Obesity is widely dispersed geographically. These data indicate the need for obesity prevention strategies that consider both the environmental and individual factors. As obesity can lead to many life-threatening complications, it is vital to have national education and prevention programs. There is a need to map obesity in Iranian children and adults, and conduct meta-analyses based on geographic and climatic regions. To prevent future obesity epidemics, a massive international program must be designed.

## Data Availability

The datasets used and/or analyzed during the current study are available from the corresponding author on reasonable request.
